# Malignant peripheral nerve sheath tumours in inherited disease

**DOI:** 10.1186/2045-3329-2-17

**Published:** 2012-10-04

**Authors:** D Gareth R Evans, Susan M Huson, Jillian M Birch

**Affiliations:** 1 Genetic Medicine, The University of Manchester, Manchester Academic Health Science Centre, Central Manchester Foundation Trust, St. Mary’s Hospital, Oxford Road, Manchester, M13 9WL, UK; 2 Cancer Research UK Paediatric and Familial Cancer Research Group, University of Manchester, Room 1.900 Stopford Building, Oxford Road, Manchester, M13 9PT, UK; 3 Manchester Academic Health Science Centre, Genetic Medicine, St Mary’s Hospital, Central Manchester Hospitals Foundation Trust, Manchester, M13 9WL, UK

**Keywords:** Sarcoma, MPNST, NF1, Li Fraumeni, TP53, SMARCB1, NF2

## Abstract

**Background:**

Malignant peripheral nerve sheath tumours (MPNST) are rare tumours known to occur at high frequency in neurofibromatosis 1 (NF1), but may also occur in other cancer prone syndromes.

**Methods:**

The North West Regional Genetic Register covers a population of 4.1 million and was interrogated for incidence of MPNST in 12 cancer prone syndromes. Age, incidence and survival curves were generated for NF1.

**Results:**

Fifty two of 1254 NF1 patients developed MPNST, with MPNST also occurring in 2/181 cases of schwannomatosis and 2/895 NF2 patients. Three cases were also noted in *TP53* mutation carriers. However, there were no cases amongst 5727*BRCA1/2* carriers and first degree relatives, 2029 members from Lynch syndrome families, nor amongst 447 Familial Adenomatous Polyposis, 202 Gorlin syndrome, nor 87 vHL cases.

**Conclusion:**

MPNST is associated with schwannomatosis and *TP53* mutations and is confirmed at high frequency in NF1. It appears to be only increased in NF2 amongst those that have been irradiated. The lifetime risk of MPNST in NF1 is between 9–13%.

## Introduction

Malignant peripheral nerve sheath tumour (MPNST) are uncommon tumours varying substantially in clinicopathologic features [1]. Previous studies have shown that 20%–50% of patients with MPNST also have Neurofibromatosis type 1 (NF1) [2-4]. Cutaneous MPNST is very rare [5-7]; skin involvement is usually secondary to local invasion or metastasis from larger underlying tumours. Most are high grade, poorly differentiated and aneuploid. Only half can be shown to exhibit schwannian differentiation by immunohistochemical methods. Tumours exhibiting mesenchymal primarily rhabdomyosarcomatous differentiation (Triton tumour) are often associated with NF1 [8]. The lifetime risk of MPNST in NF1 has previously been assessed as between 8–13% [4]. However, there has been limited evidence of association with other tumour prone disorders, with only case reports showing associations with *TP53* mutations [9] and schwannomatosis [10]. We have reviewed our genetic register databases for presence of MPNST amongst affected cases and for non-syndromic conditions their first degree relatives (FDRs). The conditions studied are summarised in Table 1. 

**Table 1:** Diagnostic criteria for NF1 (two or more must be present)

**Disease**	**Birth incidence**	**Main tumour associations**	**Known risk of MPNST**
NF1	1 in 2,712	Neurofibroma, MPNST, glioma AML, phaeochromocytoma	yes
Schwannomatosis	1 in 100,000	Schwannoma, meningioma	No
NF2	1 in 33,000	Schwannoma, meningioma, ependymoma	Only after irradiation
BRCA1	1 in 900	Breast cancer, Ovarian cancer	No
BRCA2	1 in 850	Breast cancer, Ovarian cancer, prostate cancer	No
TP53	1 in 3000	Breast cancer, sarcoma, glioma, lung cancer, adreno-cortical	possible
FAP	1 in 8,619	Colorectal, duodenal, hepatoblastoma, thyroid	No
MSH2	1 in 2000	Colorectal, endometrium, ovarian, upper urothelial, gastric, glioma	No
MLH1	1 in 2000	Colorectal, endometrium, ovarian, upper urothelial, gastric, glioma	No
MSH6	1 in 10,000	Colorectal, endometrium, ovarian, upper urothelial, gastric, glioma	No
vHL	1 in 42,987	Renal cancer, haemangioblastoma	No
Gorlin syndrome	1 in 18,976	Basal cell carcinoma, medulloblastoma	No

## Methods

The North West Regional Genetic Register covers a region of North West England, based around Manchester, with a population of 4.1 million. The genetic register service covers a number of tumour predisposing syndromes in particular NF1, Neurofibromatosis type 2 (NF2), Familial Adenomatous Polyposis (FAP), Gorlin syndrome, von Hippel Lindau disease and non-syndromic families with *BRCA1, BRCA2, MSH2, MLH1* and *MSH6* mutations. The register is highly ascertained for NF1, NF2, FAP, Gorlin syndrome von Hippel Lindau disease [11]. In addition there is a regional database of *TP53* mutation carriers and an international database for schwannomatosis patients referred into the Manchester laboratory for *SMARCB1* mutation analysis. All the registries have been the subject of cancer verification using the regional North West Cancer Intelligence Service (NWCIS). We have previously published risks of cancers in Lynch syndrome [12] and cancer incidence in *BRCA1* and *BRCA2* carriers [13]. The NWCIS ascertains patients with malignancies of all sites, as well as benign central nervous system tumours, from pathology records and death certificates. We previously reviewed the Cancer Register for patients with MPNST (ICD-0: M9540/3 and 9560/3) from 1984–1996 [4]. We reviewed hospital notes for all patients with MPNST who were identified for the study. To corroborate a diagnosis of NF1, histology reports and details of other typical NF1 disease features were reviewed (e.g., café-au-lait macules and neurofibromas with respect to the U.S. National Institutes of Health diagnostic criteria [11]). As a diagnosis is straightforward in most cases of NF1, NF2, vHL, Gorlin syndrome and FAP only affected cases were used in the analysis. As previously we also used all FDRs of known mutation carriers for Lynch syndrome (*MSH2, MLH1, MSH6*) [12] and *BRCA1/2*[14] as 50% of these are likely to carry the pathogenic mutation.

Death details from the registries were used to calculate survival rates, and death certificates were reviewed to determine cause of death. Follow-up was censored on 1^st^ April 2010. MPNST incidence curves were derived for NF1 for strict regional residents to avoid ascertainment bias. Five-year survival was determined using Kaplan-Meier curves. NF1 MPNST cases who were identified on the periphery of the region during the study period were included for the survival analysis only. The Mann–Whitney *U* test and Wilcoxon (Gehan) statistic were used to test between-group differences in age at diagnosis and survival.

## Results

The presence of MPNST among the study populations is shown in Table 2. There were no instances of MPNST amongst 5727 *BRCA1/2* carriers and first degree relatives, nor among 2029 Mismatch Repair mutation carriers and first degree relatives from Lynch syndrome families. As the annual rate of MPNST is about 1.25 per million [4] the lifetime risk in the general population would be about 1 in 10,000. There could be some confidence therefore that MPNST does not occur at increased incidence in *BRCA1/2* carriers but not yet in Lynch syndrome. There were also no cases amongst vHL, Gorlin or FAP patients although numbers were smaller. The presence of two male cases with MPNST aged 32 (retro-orbital) and 33 years (lower limb) and one female case of triton tumour aged 5 years amongst carriers of a 524 G > A (Arg175His), a 574delC and a 375 G > A *TP53* mutation respectively suggests a likely association with Li Fraumeni syndrome. The 375 G > A mutation affects the invariant splice donor site CG/g > CA/g in exon 4. The missense and splicing mutation were inherited, but the framshift deletion *de novo.* There were also a further three benign nerve sheath tumours in three further unrelated *TP53* mutation carriers aged 33 years (Vestibular schwannoma) and two paraspinal/extra-dural at ages 50 and 49 years. There were also three cases with schwannomatosis and MPNST. Two of these occurred in a family with a c.846 C > G, p.N288K *SMARCB1* mutation that we have previously reported as probably pathogenic [15]. It appears this Australian family has been reported as part of a hospital series, but they were not noted to have a *SMARCB1* mutation [10]. Both had high grade MPNSTs with one now metastatic aged 27 years and the other having died aged 17 years. A third case in an individual with multiple spinal (5) schwannomas and a peripheral schwannoma occurred aged 46 from which he died. *SMARCB1* mutation analysis was not possible. There were two cases occurring amongst 921 NF2 patients. Both of these have been reported as having occurred after radiation treatment for vestibular schwannoma [16,17]. No MPNSTs occurred in unirradiated NF2 patients. 

**Table 2:** Incidence of MPNST in 12 tumour prone genetic conditions

**Disease**	**Cases at risk**	**MPNST**	**MPNST after radiation treatment**	**Sex ratio** **M:F**	**Median (range) age at diagnosis**
NF1	1252	52	4	22:30	31 (13.3–77.2)
Schwannomatosis	181	3	0	2:1	26 (17–45)
NF2	920	2	2	1:1	25–34
BRCA1	2992*	0	0		
BRCA2	2735*	0	0		
TP53	221*	3	0	2:1	32 (1–33)
FAP	477	0	0		
MSH2	1054*	0	0		
MLH1	897*	0	0		
MSH6	178*	0	0		
vHL	87	0	0		
Gorlin syndrome	202	0	0		

*Cases include first degree relatives of known mutation carriers.

There were 52 cases of MPNST amongst our NF1 patients including 43/1059 patients within the strict regional.boundaries of the Cancer Register. Fifteen cases of MPNST have occurred in the strict regional population since 1996 maintaining an incidence rate of above 1 per 1000 NF1 patient per year as 1010 NF1 patients were alive at some point after 1996.

### *NF1 MPNST lifetime risk*

**Figure 1:**
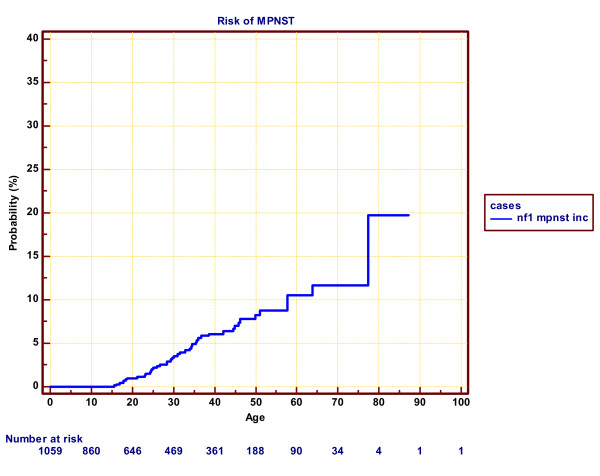
**Cumulative incidence of MPNST in NF1 patients within the strict North West region boundaries**.

Within the strict regional population of 1059 individuals the cumulative risk of MPNST was 11.7% (95% CI 9.7–13.7%) by age 70 years (figure 1). The robustness of incidence beyond 70 years is influenced by a single case in a female aged 77 years in a population with only 34 patients living beyond 70 years.

## Discussion

The association between MPNST and NF1 has long been documented [2-4,18-22]. The present report doubles the size of our previous report of MPNST in NF1 [4] and provides further confirmation for the lifetime risk estimates. As only cases with MPNST within the strict highly ascertained borders of the region are included these remain robust. There have been fifteen MPNSTs in our regional population in the fourteen years since our last population based study [4]. The annual incidence of above 1 per 1000 NF1 patients has been maintained over now a 27-year period. The lifetime risk of MPNST in NF1 is estimated at 11.7% with tighter confidence intervals than previously. Survival remains poor in NF1 with only one third of patients alive at 5 years post diagnosis. This is in accordance with our previous publication [4]. Although we have also recently reported an improved survival in recent years (since 1996) and significantly better survival in females versus males (5-year survival 46% vs 22%) with NF1 [23]. MPNST remains the single biggest contributing factor to reduced life expectancy in NF1 [16,18,24].

In addition to the known link with NF1 this study has confirmed probable links with schwannomatosis and germline *TP53* mutations. The presence of three proven MPNSTs in only 181 schwannomatosis patients is very suggestive of an association. The main difficulty with this association is the previous problems with classifying schwannomatosis [25-27]. However, the presence of two MPNSTs in a family with a proven *SMARCB1* mutation suggests that this is a real link. It also broadens the tumour spectrum in families with *SMARCB1* mutations. There remains a question as to why children with certain *SMARCB1* mutations have a very high risk of the highly Malignant atypical teratoid Rhabdoid tumours [28] whereas other families appear to get only schwannomas [15] although meningiomas also occur at a variable frequency [15,29,30].

The presence of three confirmed *TP53* mutation carriers with MPNST makes a link with germline *TP53* mutations and Li Fraumeni syndrome very likely. It is quite possible that cases of MPNST are buried amongst reports of soft tissue sarcoma [31]. It is therefore likely that occult/unrecognized LFS patients/families could come to light from performing *TP53* analysis on MPNST patients. Both the cases of MPNST occurred after the previous report from our group [31]. Nonetheless the only clear report of a *TP53* mutation was a case of Triton tumour in a three year old [9]. This report also suggests that this was the first such case in the literature whereas the further triton tumour case in our series was evident from our previous report [31].

The link between NF2 and MPNST is controversial [16,17,32-35]. There have been a number of reports of MPNST following radiation therapy [16,17,33] in NF2, but only two reports of apparently spontaneous MPNST without radiotherapy [34,36]. The first report is, nonetheless, questionable as it describes multifocal MPNST in addition to neurofibromata, which would be more consistent with NF1 than NF2. The second describes a patient with a unilateral vestibular schwannoma that developed contralateral cranial MPNST [36] as such neither report refers to a patient with proven NF2. The first report [34] also describes the possibility of a constitutional *TP53* mutation, which in addition to an NF1 mutation may cause a very substantial risk of MPNST. Indeed *TP53* mutations have been described in the transformation from benign schwannoma to MPNST [32]. Although the risk of MPNST appears to be higher in *TP53* mutation carriers this will need to be confirmed in larger studies.

Up to 50% of malignant peripheral nerve sheath tumours in non NF1 patients harbour NF1 mutations [37]. There is a broad spectrum of NF1 mutations in MPNST and in the germline of those NF1 patients that develop MPNST [38,39]. Although the risk of MPNST appears to be substantially higher in individuals with large deletions removing the NF1 gene [40], these patients still only make up a small proportion of all cases of MPNST in NF1 [38,39]. A better test needs to be derived to target a high risk population in NF1 for screening. It is possible that assessing whole body tumour burden and particularly the extent of large deep seated tumours at age 15–20 years may detect a sub-population at enhanced risk [41]. Whilst MRI can detect volumetric changes that may presage malignant change, Positron Emission Tomography (PET) is the most sensitive and specific test to determine if a tumour has become malignant [42,43]. Nonetheless the significant dose of radiation involved in PET means this should not be used in routine screening in a cancer prone syndrome [16]. Detection of a subset at risk by genetic analysis and whole body MRI followed by regular MRI of suspicious lesions for increased volumetric growth alongside greater patient awareness may improve survival from the current poor levels in NF1.

The poor survival from MPNST particularly in NF1 patients highlights the need for therapies targeted at the main underlying genetic abnormality. Whole genome sequencing is likely to reveal new targets for therapy and the fast reducing cost of such testing may mean that it will be affordable in the clinic within the next 5 years.

In conclusion MPNST appears to occur at increased frequency in schwannomatosis and in those with germline *TP53* mutations as well as those with NF1. Radiation treatment particularly in childhood increases the risk of MPNST in NF1 and may also cause MPNST to occur in NF2.

## Competing interests

The authors declare that they have no competing interests.

## Authors’ contributions

The research and reviews conceived by DGE, initial manuscript and data assessed by DGE, contributions for data on TP53 from JMB and from SMH for NF1. All authors developed the manuscript and approved the final version.

## Author details

^1^Genetic Medicine, The University of Manchester, Manchester Academic Health Science Centre, Central Manchester Foundation Trust, St. Mary’s Hospital, Oxford Road, Manchester, M13 9WL, UK. ^2^Cancer Research UK Paediatric and Familial Cancer Research Group, University of Manchester, Room 1.900 Stopford Building, Oxford Road, Manchester M13 9PT, UK.^3^Manchester Academic Health Science Centre, Genetic Medicine, St Mary’s Hospital, Central Manchester Hospitals Foundation Trust, Manchester, M13 9WL, UK.
